# What Does PLIER Really Do?

**Published:** 2008-08-27

**Authors:** Terry M. Therneau, Karla V. Ballman

**Affiliations:** Division of Biostatistics, Mayo Clinic College of Medicine, Rochester, MN 55905, U.S.A

## Abstract

**Motivation:**

Our goal was to understand why the PLIER algorithm performs so well given its derivation is based on a biologically implausible assumption.

**Results:**

In spite of a non-intuitive assumption regarding the PM and MM errors made as part of the derivation for PLIER, the resulting probe level error function does capture the key characteristics of the ideal error function, assuming MM probes only measure non-specific binding and no signal.

## Introduction

The PLIER (Probe Logarithmic Intensity ERror) algorithm was developed by Affymetrix and released in 2004. It is part of several commercially available software packages that analyze Genechip^®^ data such as Strand Genomic’s Avadis and Stratagene’s ArrayAssist^®^. The PLIER algorithm produces an improved gene expression value (a summary value for a probe set) for the GeneChip^®^ microarray platform as compared to the Affymetrix MAS5 algorithm. It accomplishes this by incorporating experimental observations of feature behavior. Specifically, it uses a probe affinity parameter, which represents the strength of a signal produced at a specific concentration for a given probe. The probe affinities are calculated using data across arrays. The error model employed by PLIER assumes error is proportional to observed intensity, rather than to background-subtracted intensity. However, the derivation of the method also assumes that the error of the mismatch probe is the reciprocal of the error of the perfect match probe. We find this assumption counter-intuitive.

**Figure 1:**
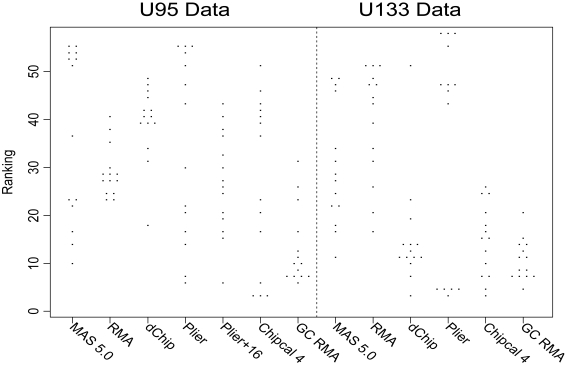
Rankings of selected methods on 14 outcomes, from the Affycomp website.

On the other hand, PLIER definitely performs well. It outperforms MAS5 in terms of the benchmark data and measures [3, 8] for assessing the quality of the summary statistic for a probe set. It also does fairly well compared to other methods that are commonly used to compute gene expression values for GeneChip probeset data. The Affycomp website (affycomp.biostat.jhsph.edu) currently shows results for 14 of the measures of accuracy [9] for 50+ normalization and analysis combinations; Figure 1 shows the ranks, on all 14 measures, for the default Affymetrix software MAS5, two early methods, robust multichip average (RMA) and dChip, and two more recent additions, chip calibration [11] and GC RMA version 1.1.3, along with those for PLIER. No one method dominates the competition in the sense of being the best or near best on all rankings, but for comparison GC RMA has the smallest average rank in the U133 data. In the U95 data, PLIER+16 does quite well, with an overall rank of sixteenth out of 54 entries. In particular, improvements over MAS5 include a higher reproducibility (lower coefficient of variation) without loss of accuracy and higher differential sensitivity for genes with lower expression values.

This inconsistency, good performance of an algorithm derived from a counter-intuitive error model assumption, prompted us to look more closely at the PLIER algorithm. Specifically, we looked at the error function for the algorithm and compared it to that for a more biologically based one. By examining the behavior of individual probes over a sequence of spiked-in RNA concentrations of a target gene, characterizations of the PLIER error function became clearer. The major finding is that the PLIER error model possesses many of the key characteristics of the ideal error function for fitting individual probe calibration curves.

## PLIER Description

This description of the PLIER algorithm is based upon a Technical Report [1] on the Affymetrix website. Consider a single probeset on an array and a set of *j* =1, 2, …, n arrays in the experiment. We assume the probeset contains *i* =1, 2, …, m probe pairs; a probe pair *i* consists of a perfect match (*PM_ij_*) and mismatch probe (*MM_ij_*). Let pm*_ij_* and mm*_ij_* represent the observed binding intensity for the perfect match and mismatch probe *i* on array *j*, respectively. The expected value for the observed binding for the perfect match and mismatch probes is assumed to be

(1) $$\begin{array}{l} E(pm_{ij})=\mu_{ij}=a_{i}c_{j}+B_{ij}\\ E(mm_{ij})=B_{ij} \end{array}$$

where

- *B_ij_* is background binding for probe pair *i* on array *j* (background is assumed to be the same for the PM and MM probes within a pair),
- μ*_ij_* is the binding level of probe *i* on array *j*,
- *a_i_* is the binding affinity of probe *i*,
- *c_j_* is the concentration of RNA in sample *j*, which is hybridized to array *j*.

The quantities *B_ij_*, μ*_ij_*, *a_i_*, and *c_j_* represent the (unknown) true values of the background binding, probe binding, affinity, and concentration, respectively, whereas *pm_ij_* and *mm_ij_* are the observed intensity values.

It is fairly well established from empirical data that the logarithm (log) of the observed binding intensities is approximately equivariant; in other words, the error is multiplicative. This implies the following model

$$\begin{array}{l} pm_{ij}=\mu_{ij}{ɛ}_{ij}^{P}\\ mm_{ij}=B_{ij}{ɛ}_{ij}^{M} \end{array}$$

where *ε^P^* and *ε^M^* are random terms for the PM and MM probes, respectively, from an appropriate distribution, a log-normal for instance. Subtracting the observed MM probe binding intensity from its corresponding PM partner yields,

$$pm_{ij}-mm_{ij}=(a_{i}c_{j}+B_{ij}){ɛ}_{ij}^{P}-B_{ij}{ɛ}_{ij}^{M}.$$

The assumption that the perfect match and mismatch error for probe pair *i* are equal, i.e. *ε_ij_^P^* = *ε_ij_^M^* = *ε_ij_*, produces

$$pm_{ij}-mm_{ij}=(a_{i}c_{j}){ɛ}_{ij},$$

which is the original MAS5 equation. The issues and limitations associated with this error model, especially for low intensities (low binding), are well known [6, 7]. PLIER does not assume that the perfect match and mismatch errors within a probe pair are equal, but rather assumes that *ε_ij_^P^* = 1/*ε_ij_^M^*. This seems counter-intuitive biologically; especially since the PM and MM probes within a given probe pair are physically adjacent to each other on the array. Any local artifact would be expected to affect both probes in the same direction rather than causing the error of one to increase when the error of the other decreases. Under the PLIER error assumption, Equations (1) can be rearranged as,

(2) $$\begin{array}{l} {ɛ}_{ij}=\frac{a_{i}c_{j}+\sqrt{{\left(a_{i}c_{j}\right)}^{2}+4pm_{ij}mm_{ij}}}{2pm_{ij}}\\ =\frac{{\hat{\mu }}_{ij}/pm_{ij}+\sqrt{{\left({\hat{\mu }}_{ij}/pm_{ij}\right)}^{2}+4(mm_{ij}/pm_{ij})}}{2} \end{array}$$

The PLIER algorithm selects *a* and *c* such that the average “residual” *r* = log(*ε*) equals zero. Specifically, this is accomplished by minimizing a robust average of the *r*^2^ values. The particular robust M-estimator used (Geman-McClure) is not of particular interest here. If the mismatch binding MM is zero, then log_2_(*μ̂_ij_*) = log_2_(*pm_ij_*) − *r_ij_*, which shows that the estimate *μ̂* is closely related to the geometric mean or log average of the PM probes. The presence of MM binding increases the estimate for μ.

**Figure 2:**
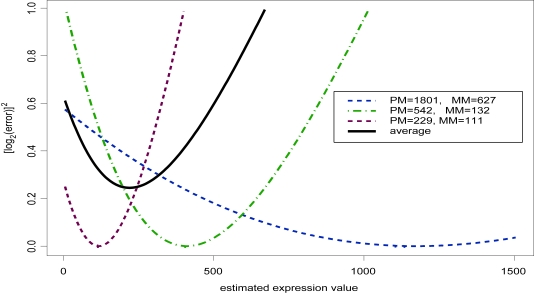
Error curves for various combinations.

To more concretely understand how this algorithm works, consider a case of a single probeset on a single array. The goal is to obtain an estimate of the gene expression value for the probeset. For simplicity, assume there are only 3 probe pairs in the probeset. In this example, we use the first three probes of the U95A probeset 37777 at where the corresponding transcript of the target gene, protein tyrosine phosphatase receptor B (PTPRB), was spiked into a background of human pancreas RNA at a concentration of 32 PM. The observed (*pm, mm*) intensity pairs were: (1801,627), (542, 132), and (229, 111). Figure 2 displays the *r*^2^curves for these probes as a function of the estimate for the true intensity (μ*_ij_*= *a_i_c_j_*), as well as the average *r*^2^ across all three probesets. Average probeset error is minimized by an estimate of 220 as the the true expression level of this gene. The argument is similar for the complete probeset of 16 probe pairs; the plot would just be more crowded.

## The Direct Argument

### Spike-in data

**Figure 3:**
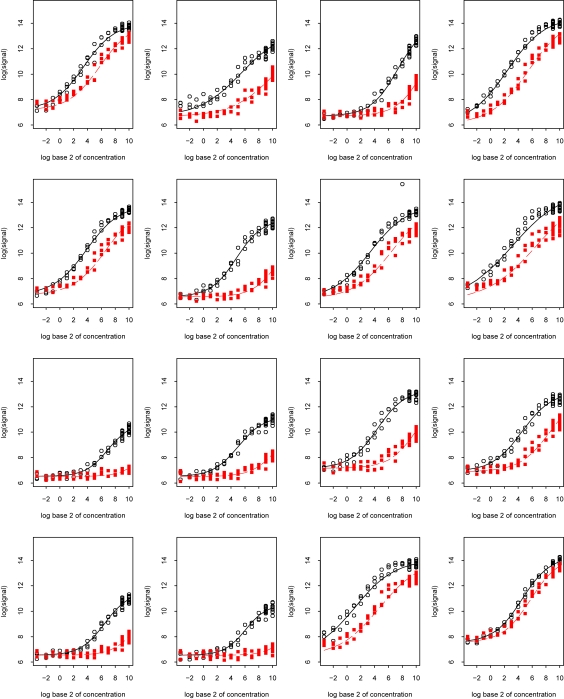
The probe pair intensity values versus the spiked concentration of a spiked transcript, probeset 37777 at, from the Affymetrix U95A spike-in experiment. There are 16 probe pairs. The open circles are the values of the PM probes and the filled squares are the values of the MM probes. The solid and dashed lines correspond to the fitted logistic calibration curves for the PM probes and MM probes, respectively.

To better understand why PLIER does well, we begin by examining characteristics of the Affymetrix data. A spike-in experiment dataset was created by Affymetrix and is publicly available at their web site www.affymetrix.com; search on the phrase “Latin square data” to find the link to the page containing a description of the experiment and the downloadable files of data. In this experiment, mixtures of a common RNA background, in which 16 probesets were spiked in according to 14 different concentrations (0, 0.25, 0.5, 1, 2, 4, …, 512, 1024 PM), were hybridized to a set of Affymetrix U95Av2 arrays. In most cases, each pattern of the 16 probeset concentrations was replicated three times. A cyclic latin square design was used for the spike-in pattern of the target RNAs. Irizarry et al. [7] provide a more detailed description of this experiment. Figure 3 contains the plots of the probes within the first spiked-in probeset 37777 at. Each plot contains the observations of the perfect match and mismatch probes. The probeset 37777 at was spiked in at 14 different concentrations (0, 0.5, 1, …, 1024 PM) across a total of 59 arrays. The observed expression values were plottedonthe *y*-axis and the spike-in concentrations were plotted on the *x*-axis; both on a log_2_ scale. (The data has not been normalized; however, this particular plot is almost unchanged by normalization.) A panel is shown for each probe in the probeset; the perfect match (PM) and mismatch (MM) values were plotted using different symbols. Fitted S shaped curves were superimposed on the data, where the PM function differed from the MM function only in the location of its infection point; a paper by Ballman and Therneau [2] contains the complete set of plots for this and other spike-in experiments. As can be seen in Figure 3, the S-shaped curves appear to fit the data well.

### Models of the data

From the literature, there are at least two data models appropriate for the Affymetrix data. If we assume that binding to the chip surface (probeset) does not change the concentration of the target (cDNA) in solution, then the standard mass action laws lead directly to the Langmuir isotherm equation. Its appropriateness for modeling Affymetrix data is described nicely by Hekstra et al. [5]. Let *x* be the specific RNA concentration, then the fraction of occupied probe sites θ is given by

$$\theta =\frac{2^{x}}{2^{x}+k}$$

where *K* is the concentration at which half the surface sites are occupied; *K* is a function only of the binding affinity of the probe. Assuming the measured fluorescence intensity to be linearly dependent on the amount bound to the probe, we get the following model for the intensity *y*

(3) $$y=b+d\theta =b+d\frac{2^{x}}{2^{x}+k}$$

where *b* and *d* have units of intensity. The model predicts chemical saturation at *b* + *d* for high concentrations of RNA. It can also be shown that competitive cross-hybridization by non-specific RNAs in the target solution does not change the functional form of Equation 3 but only affects the parameter values.

The second model was described by Finney [4] for behavior of calibration curves of radioligand assays where *x* is the log of the (known) dose and *y* the log of the observed intensity from the assay. Finney suggested that for this, and most binding equations, a logistic or probit function adequately describes the relationship between *x* and *y*. As seen in Figure 3, an S-shaped curve such as a logistic appears appropriate because it captures the effect of background binding and/or lower limits of detection (i.e. the flat lower portion of the lefthand part of the curve) and the effect of biochemical saturation and/or the instrumentation (i.e. the upper portion of the righthand part of the curve).

**Figure 4:**
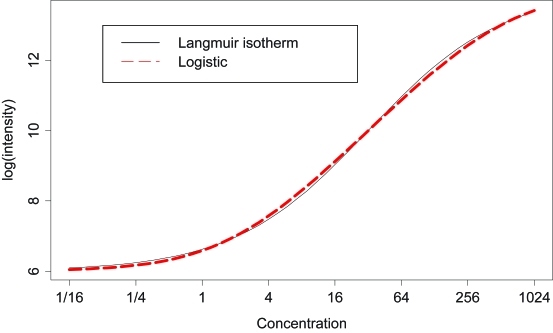
A logistic curve and Langmuir isotherm curve.

Is one of these models more appropriate than the other? Figure 4 shows a logistic curve and a Langmuir isotherm curve, both scaled to the range of data values in Figure 3. Clearly, the two curves are virtually indistinguishable. In light of this, we fit logistic curves to the PM and MM data in Figure 3. The logistic curves were fit simultaneously where the PM curve only differed from the MM curves in the location of the infection point, i.e. assuming the same upper and lower thresholds *a* and *b* but a different binding affinity *K*. The PM and MM curves have identical shapes but the MM curve is shifted to the right of the PM curve (lower affinity). As can be seen in Figure 3, the theoretical curves fit the data well.

### Graphical comparison of error models

The ideal estimate of gene expression for an experiment would use the probe curves from Figure 3 directly; which is unfortunately not possible since the curves are unknown. But let us assume for a moment that they were, with *f_i_*(*x*) being the calibration curve for the *i*th probe. Since the data *y* are approximately equivariant on the log scale, a rational approach for estimating the binding is to minimize the overall error

$$\begin{array}{l} E=\sum_{i}{\left[f_{i}(\hat{x})-y_{i}\right]}^{2}\\ \equiv \sum_{i}e{\left(y_{i},\hat{x}\right)}^{2} \end{array}$$

Over a set of probes, one wishes to choose the estimated binding *x* so as to make errors close to zero. (This is essentially how assays for which a binding curve is created as a part of the procedures, such as ELISA, proceed.)

To combine PM and MM values, our ideal error function will be the simple sum, (*f_pm_*(*x̂*)−log_2_ (*pm*)) +(*f_mm_*(*x̂*)−log_2_(*mm*)). The error function used by PLIER is shown in Equation 2. In MAS5, the binding estimate is an average of the log_2_(*pm*−*mm*) differences, which is equivalent to the linear error function *x̂*−log_2_(*pm*−*mm*).

**Figure 5:**
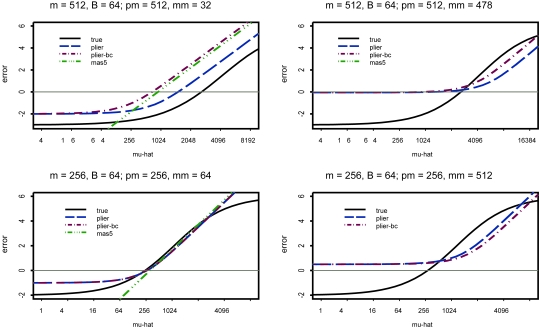
The functions for the ideal error (true), the MAS5 error (mas5), PLIER error(plier), and PLIER applied to background-adjusted data error(plier-bc). The true gene intensity (*μ*) and background (*B*) and the observed *pm* and *mm* values are on each plot.

All other functions can be compared to this ideal error function. We compare the error functions for two different true concentrations for a probe, μ = 512 and μ = 256 (9 and 8 on the log_2_ scale, respectively), with a known binding background level of 64 (6 on the log_2_ scale). Specifically, we compare the error as a function of the estimated μ, i.e. *μ̂*, values under the MAS5 model and PLIER model to the ideal error curve (from S-shaped calibration curve). The error functions for MAS5 and PLIER are a function of the observed *pm* and *mm* values. The PLIER (and MAS5) functions presented were applied to non-background adjusted data. However, PLIER (and MAS5) is applied to global background adjusted data in practice and so we show the error curve for PLIER applied to background-adjusted data. The global background adjustment we used was 64, which roughly corresponds to the 0.02 quantile of all the probe values (this is the default global background correction of MAS5). Figure 5 shows the form of the error functions on the same plot for different observed values of the *pm* and *mm* values. Note that these error functions are idealized in that they have been shifted so that they all have the smallest error (zero error if possible) at the true binding intensity value. The amount of shift necessary differs for the different functions and would be unknown in practice. Hence, this is a comparison of errors under perfect conditions for each function.

As can be seen from the panel of plots, the implied error function for MAS5 differs dramatically from the ideal error function in the lower tail. This explains the poor behavior of MAS5 for estimating expression values for low RNA concentration levels, which has been cited extensively in the literature. Also note that there is no curve for the MAS5 model when *pm* = 256 and *mm* = 512. The reason is that when the *mm* value is larger than the *pm* value, the expression value is undefined. This is not technically true for MAS5 because in instances where *pm* < *mm*, the algorithm employs an ideal mismatch value; the ideal mismatch value is selected such that it is less than the *pm* value. However, when *pm* > *mm*, which occurs for the majority of the probe pairs, the error functions in Figure 5 are correct.

On the other hand, the implied error curve for the PLIER model has the correct shape for the left portion of the function. This explains the observation that PLIER yields improved estimations of expression values for low RNA concentration levels compared to MAS5. Neither PLIER nor MAS5 error functions have the correct shape for the right-hand portions of the plots. In practice, the effect of differences from the ideal error for the far right portion of the function is not as serious as differences in the left portion. This is because for actual experiments employing collected biospecimens of interest (cell lines, animal tissue, or human tissue), saturation of the probes is rarely reached. However, when the MM value is far above background, as it is for the spike in experiment when the observed MM values are greater than 256, the overly high lower threshold of the PLIER error function can cause overestimation. Finally, PLIER applied to global background adjusted data does not perform as well as PLIER applied to unadjusted data. There are several variations of PLIER, e.g. PLIER+16 and PLIER+32, which add the constants 16 and 32, respectively, back to global background adjusted data. These constants are on the order of magnitude of the values that were subtracted for global background adjustment and largely “undo” the global background adjustment; from above, we see that PLIER performs better on data that has not been globally background adjusted.

## Properties of the Error Function

From the graphical display of the error functions for MAS5 and PLIER, it appears as though a possible explanation for why PLIER performs so well is that in the crucial part of the error function, it has characteristics similar to the ideal error curve. What are the general characteristics of the ideal error function?

Assume that the true assay binding calibration function is a logistic curve, or something quite like it, so

$$\text{log}(pm_{ij})=f(\mu_{ij})+{ɛ}_{ij}$$

with *ε* from a symmetric distribution, which is equivariant across the range of the data. The true concentration μ*_ij_* will be estimated with a model of interest such as array + probe effects. A rational approach for estimating the parameters is to minimize the overall sum errors of Equation 4. Figure 5 shows some specific examples; can we describe the behavior more generally?

Let us assume that *f* has a lower threshold or background, log_2_(*b*), which corresponds to the scanner effect and non-specific binding when the target gene is not expressed. To the right of this threshold, assume *f* is linear or nearly linear on the log_2_ scale, and is smooth. Under these conditions, the error function would have the following properties.

1. For *μ̂_ij_* small, *e* → log(*pm_ij_*) − log(*b*) = log (*pm_ij_*_/_b).
2. For *μ̂_ij_* large enough so that *f*(*uij*) is in the linear part of the curve (i.e. sufficiently larger than log(*b*)), the derivative of *e* with respect to *uij* will be a constant.
3. The behavior described in 2 is independent of the value of *pm*_ij_.

For PLIER, we can verify 1 and 2 above, algebraically; this confirms the behavior observed in Figure 5 for the general case.

For property 1, the error in Equation (2) is placed on the log_2_ scale and multiplied by −2 to get ε^*^

$$\begin{array}{l} {ɛ}^{*}=-2\times {\text{log}}_{2}(ɛ)\\ =-2\times {\text{log}}_{2}\\ \left[\frac{\mu_{ij}/pm_{ij}+\sqrt{{\left(\mu_{ij}/pm_{ij}\right)}^{2}+4(mm_{ij}/pm_{ij})}}{2}\right] \end{array}$$

As μ*_ij_* → 0 (so *μ̂_ij_* → 0),we get

$$\begin{array}{l} {ɛ}^{*}\to -2\times {\text{log}}_{2}\left[\frac{0+\sqrt{0+4(mm_{ij}/pm_{ij})}}{2}\right]\\ \to {\text{log}}_{2}(pm_{ij})-{\text{log}}_{2}(mm_{ij})={\text{log}}_{2}(pm_{ij}/mm_{ij}) \end{array}$$

Under the Affymetrix assumption, the PM probe measures the target gene concentration and the MM probe measures the background level. Since *pm* estimates the signal level and mm estimates MM, or background, respectively, this satisfies the first property. Note, it has been established that MM does not measure background alone but also measures signal. However, as the true concentration level, μ, becomes small, MM becomes a better estimate of background, i.e. it is less likely to also measure signal. Hence, the PLIER error function is reasonably consistent with property 1.

For property 2, we again place the error on the log_2_scale, drop the subscripts, and we get

$${ɛ}^{*}={\text{log}}_{2}\left[\frac{\hat{\mu }/pm+\sqrt{{\left(\hat{\mu }/pm\right)}^{2}+4(mm/pm)}}{2}\right]$$

Now we take the derivative with respect to log_2_ (*μ̂*)

$$\begin{array}{c} \frac{d{ɛ}^{*}}{d{\text{log}}_{2}(\hat{\mu })}=\left(\frac{1}{\text{ln}2}\right)\\ \left(\frac{1}{\hat{\mu }/pm+\sqrt{{\left(\hat{\mu }/pm\right)}^{2}+4(mm/pm)}}\right)\\ \left(\frac{1}{pm}+\frac{\hat{\mu }/pm^{2}}{\sqrt{{\left(\hat{\mu }/pm\right)}^{2}+4(mm/pm)}}\right)\left(\frac{\hat{\mu }}{\text{ln}(2)}\right) \end{array}$$

If we assume that background is small compared to signal (i.e. as we move away from background levels) and that *mm* is a good estimate of background, then (*mm/pm*) → 0 as *pm* increases. Under these assumptions, as *pm* increases, we get

$$\begin{array}{l} \frac{d{ɛ}^{*}}{d\hat{\mu }}=\left(\frac{1}{\text{ln}2}\right)\left(\frac{1}{\hat{\mu }/pm+\sqrt{{\left(\hat{\mu }/pm\right)}^{2}+}4(0)}\right)\\ \left(\frac{1}{pm}+\frac{\hat{\mu }/pm^{2}}{\sqrt{{\left(\hat{\mu }/pm\right)}^{2}+4(0)}}\right)\left(\frac{\hat{\mu }}{\text{ln}2}\right) \end{array}$$

So again, under somewhat reasonable assumptions, the PLIER error is consistent with the second property of the ideal error function. In addition, it is also consistent with property 3.

PLIER is of course making the assumption that log(MM) = background + error; in particular, it assumes that *mm* does not measure any gene signal. If this assumption is true, we see from above that the PLIER error model has the characteristics of the ideal error model, especially in the region of the plot that is the hardest, the low end. This explains why it does perform better than MAS5. However, the more these assumptions are violated—i.e. the more signal the observed *mm* measures in addition to non-specific binding, the more the PLIER error function will deviate from the ideal error function. As mentioned previously, it is fairly well established in the literature that mm does measure signal in addition to non-specific binding, which may explain why PLIER is not the best performing algorithm of those entered in Affycomp.

## Conclusions

In light of the fact that the MM probes are not good estimates for probe background level, the PLIER algorithm could likely be improved with a better estimate of background binding, perhaps along the lines of that proposed by Naef et al. [10]. Another question, one which we did not address here, is whether a robust average, such as that employed by PLIER, is really necessary. This is based on the fact that on a log scale, the spike-in data appear relatively equivariant, with few outliers. However, these considerations are of secondary importance. Of major concern is the fact that the error model is based upon an implausible assumption regarding the relationship between the error of the PM values and MM values.

Overall, we found that in spite of the non-intuitive assumption regarding the PM and MM errors made as part of the derivation for PLIER, the resulting model does capture the key characteristics of the ideal error curve, assuming MM probes only measure non-specific binding and no signal. Our only explanation for why this should be is good fortune.

This note has only considered the shape of the PLIER error function for a single probe. When averaging over multiple probes not only the shape but the relative shifts of the per probe error curves from one another will affect the effectiveness of the final estimate; our paper does not predict how PLIER will fair in comparison to other methods. In particular, we believe the deviations of the individual error functions from the ideal error functions likely will be compounded when performing the averaging across the probes in a probeset. Our belief is based on the observation that although PLIER performs better than MAS5, it does not perform as well as other algorithms entered in Affycomp, most of which are based on more biologically plausible assumptions.
